# Exploring the Connection Between Depression, Inflammatory Biomarkers, and Atherosclerotic Coronary Artery Disease

**DOI:** 10.3390/jcm14092946

**Published:** 2025-04-24

**Authors:** Marius Rus, Cristian Nicolae Sava, Adriana Ioana Ardelean, Georgeta Pasca, Felicia Liana Andronie-Cioara, Simina Crisan, Claudia Teodora Judea Pusta, Madalina Ioana Guler

**Affiliations:** 1 Department of Medical Disciplines, Faculty of Medicine and Pharmacy, University of Oradea, 410073 Oradea, Romania; rusmarius@uoradea.ro (M.R.); cristian.sava@didactic.uoradea.ro (C.N.S.); 2 Cardiology Department, Bihor Clinical Emergency Hospital, 410169 Oradea, Romania; 3 Department of Preclinical Disciplines, Faculty of Medicine and Pharmacy, University of Oradea, 410073 Oradea, Romania; adriana_ardelean@uoradea.ro; 4 Department of Psycho Neuroscience and Recovery, Faculty of Medicine and Pharmacy, University of Oradea, 410073 Oradea, Romania; spasca@uoradea.ro (G.P.); fcioara@uoradea.ro (F.L.A.-C.); 5 Cardiology Department, “Victor Babes” University of Medicine and Pharmacy, 2 Eftimie Murgu Sq., 300041 Timisoara, Romania; simina.crisan@umft.ro; 6 Department of Morphological Disciplines, Faculty of Medicine and Pharmacy, University of Oradea, 410073 Oradea, Romania; 7 Faculty of Medicine and Pharmacy, University of Oradea, 410073 Oradea, Romania; gulermadalina0106@gmail.com

**Keywords:** depression, inflammatory biomarkers, CRP, fibrinogen, IL-6, TNF-α, cortisol, acute coronary syndrome, cardiovascular diseases

## Abstract

**Background/Objectives:** Depression is associated with an increased risk for the development and progression of cardiovascular disease. This research investigated the association between depressive symptoms and inflammation in the development of atherosclerotic coronary events. **Methods:** This retrospective observational study included 276 patients who were not previously diagnosed with atherosclerotic coronary artery disease at the beginning of the research. Participants were categorized using the Hamilton Depression Rating Scale (HDRS) and the Structured Clinical Interview for DSM-5 (SCID) into two groups: the depression group and the control group. Inflammatory biomarkers (C-reactive protein (CRP), fibrinogen, interleukin-6 (IL-6), tumor necrosis factor-α (TNF-α), and cortisol) were measured at the beginning of the study, as well as at six months, one year, and two years. **Results:** Among patients with mild depression (17.3% vs. 4.2%) or moderate depression (15.4% vs. 6.7%), there were significantly more men than women, while among patients with very severe depression, there were significantly more women than men (21.7% vs. 11.5%). Participants with depression showed significantly higher increases at 2 years compared to baseline for all investigated parameters (*p* < 0.001). Depressed patients were significantly associated with an acute coronary syndrome (*p* = 0.038). **Conclusions**: This research highlights that individuals with depression face a greater risk of developing an acute coronary syndrome than those without depression.

## 1. Introduction

By 2030, depression is expected to become the leading cause of disability worldwide, significantly contributing to the global disease burden. Cardiovascular diseases (CVD) and depression are both major global health issues, and their interplay significantly contributes to premature mortality, disability, and a reduced quality of life. These two conditions are closely linked and often exacerbate each other, leading to worse health outcomes for individuals affected by both. Abnormalities in endothelial function, hypothalamic–pituitary–adrenal (HPA) axis regulation, and platelet activity have been identified as key pathophysiological links between depressive disorders and cardiovascular disease. Impaired endothelial function has been linked to depression, resulting in diminished nitric oxide production and reduced vasodilatory capacity. This vascular dysfunction plays a role in the pathogenesis of atherosclerosis and elevates the risk of cardiovascular events. Chronic depression can lead to sustained activation of the HPA axis, resulting in elevated cortisol levels. This hormonal imbalance adversely affects cardiovascular health by promoting inflammation, increasing blood pressure, and contributing to metabolic disturbances. Depressed individuals often exhibit increased platelet activation, leading to a hypercoagulable state. This heightened platelet reactivity enhances the risk of thrombosis, potentially precipitating acute cardiovascular events such as heart attacks and strokes. Inflammation is a key driver in atherosclerotic plaque formation, linking cardiovascular disease onset to the upregulation and release of intercellular adhesion molecules (ICAMs), especially soluble ICAM-1. This process is accompanied by both local and systemic elevation of inflammatory biomarkers such as interleukin-6, tumor necrosis factor, and C-reactive protein. Adhesion molecules and inflammatory mediators act as peripheral biomarkers of vascular wall inflammation [1] and have been strongly linked to the development of atherosclerosis [2] and an elevated risk of myocardial infarction and stroke [3]. Recognition of depression as a major risk factor for adverse cardiac events has been formally emphasized by the American Heart Association [4].

## 2. Materials and Methods

### 2.1. Study Design and Sampling

A total of 276 patients (120 women and 156 men) were included in this retrospective observational study between March 2022 and March 2024. The sample included patients who met the predefined inclusion and exclusion criteria relevant to the study objectives.

### 2.2. Ethical Considerations and Data Management

This study was approved by the Ethics Committee of the Bihor County Clinical Emergency Hospital, and all procedures adhered to the ethical standards outlined in the Declaration of Helsinki. Upon hospital admission, all patients signed written informed consent, which included permission for their anonymized medical data to be used for research purposes. Only data from patients who had granted this general research consent were included in the study. All data were de-identified prior to analysis to ensure confidentiality. Regarding vulnerable populations, such as individuals with severe depression, we acknowledge the ethical responsibility to ensure their protection. As this study was retrospective, no direct interventions were performed. However, we confirm that all patients included had access to standard psychiatric and medical care during the study period, and no data were used that could pose any risk to individual privacy or well-being. Missing data and attrition were addressed through a structured data cleaning process. Only patients with complete data on depression assessments and inflammatory biomarkers over the 2-year follow-up were included in the final analysis.

### 2.3. Participant Selection

Participants were retrospectively selected from hospital archive based on predefined eligibility criteria. This approach, while common in retrospective designs, may still introduce some risk of selection bias; however, efforts were made to minimize this by applying consistent criteria across the entire study period.

Participants were eligible for inclusion if they met the following criteria: ages between 18 and 80 years; no prior diagnosis of atherosclerotic coronary artery disease (CAD), including myocardial infarction, angina, or any coronary revascularization procedures. To minimize potential confounding influences on inflammatory biomarker levels, individuals were also required to be free from acute infections or chronic inflammatory conditions and not receiving immunosuppressive or anti-inflammatory medications. All participants had to be capable of providing informed consent and willing to complete standardized psychological assessments and blood sampling procedures.

Exclusion criteria included age below 18 or above 80 years and a known history of coronary artery disease. Individuals diagnosed with chronic inflammatory or autoimmune diseases (e.g., rheumatoid arthritis, systemic lupus erythematosus, or inflammatory bowel disease) were excluded due to their potential impact on systemic inflammatory markers. Additional exclusions included active or recent (within the past three months) infectious illness or inflammatory conditions, current or recent cancer (within the past five years, with the exception of non-melanoma skin cancers), pregnancy or lactation, and recent major surgery or trauma. Individuals with severe psychiatric disorders (e.g., schizophrenia, bipolar disorder), current substance use disorder, or those unable or unwilling to provide informed consent were also excluded. Patients with incomplete or inconsistent data were excluded.

These criteria were selected to ensure the study sample consisted of individuals without pre-existing cardiovascular conditions or systemic factors that could independently affect inflammatory or psychological status.

### 2.4. Baseline Characteristics of the Patients

Baseline characteristics, including age, gender, and provenience (urban vs. rural), were carefully documented and compared between groups to ensure demographic balance. Comorbidities such as hypertension or diabetes were intentionally excluded as our primary aim was to isolate the potential role of depressive symptoms and inflammatory biomarkers in the onset of acute coronary events, independent of traditional cardiovascular risk factors (Table 1).

A total of 136 patients (49.27%) were diagnosed with depression using the Hamilton Depression Rating Scale (HDRS) and the Structured Clinical Interview for Statistical Manual of Mental Disorders, Fifth Edition (SCID DSM-5), while the remaining 140 patients (50.73%) were included in the study with no symptoms and signs of depression. The HDRS is a well-established and extensively utilized tool in both clinical practice and research for assessing depression. It includes 21 items that cover a broad range of depressive symptoms, such as low mood, feelings of guilt, suicidal ideation, sleep disturbances, and anxiety. Depending on the item, responses are rated on a scale from either 0 to 2 or 0 to 4, reflecting the severity of each symptom. Total scores are typically interpreted as follows: 0–7 indicates no depression, 8–13 mild, 14–18 moderate, 19–22 severe, and scores above 23 suggest very severe depression. This cumulative score helps clinicians evaluate the intensity of depressive symptoms and supports decision-making regarding diagnosis and treatment planning [5,6]. To ensure diagnostic precision and reduce the risk of misclassification due to somatic symptoms that overlap with cardiovascular disease, all participants who screened positive for depressive symptoms underwent a structured clinical interview using the SCID DSM-5 [7]. Participants were considered to meet diagnostic criteria if they exhibited a minimum of five characteristic symptoms over a continuous two-week period, signifying a deviation from previous psychological functioning. One of the core symptoms—either persistent low mood or a marked loss of interest or pleasure in most activities—was required. Additional symptoms included disturbances in appetite or weight, sleep irregularities (insomnia or hypersomnia), psychomotor agitation or retardation, reduced energy levels, excessive guilt or feelings of worthlessness, impaired concentration, and recurrent thoughts of death or suicide. These symptoms had to cause significant emotional or functional impairment and could not be attributed to the physiological effects of a medical condition or substance use. Clinical interviews were conducted by trained mental health professionals using the SCID DSM-5 Disorders—Clinician Version (SCID-5-CV) to confirm the diagnosis [8].

The diagnosis of acute coronary syndrome was established through clinical and paraclinical investigations. The main symptoms were: sensation of pressure, tightness, or pain in the chest. These symptoms were often accompanied by discomfort that radiated to the shoulders, arms, neck, jaw, or back and, in some cases, were associated with shortness of breath, sweating, nausea, or dizziness. Paraclinical investigations included electrocardiography (ECG) to assess electrical activity and detect ischemic changes, arterial blood pressure measurement to monitor hemodynamic status, and laboratory analyses, with a focus on high-sensitivity troponin for detecting myocardial injury. Additionally, transthoracic echocardiography was performed to evaluate cardiac function, wall motion abnormalities, and potential complications, while coronary angiography was performed to detect occlusions and enable stent placement and ensuring the restoration of adequate blood flow.

Venous blood samples were collected at the beginning of the study, as well as at six months, one year, and two years. Several biomarkers were measured, including C-reactive protein (CRP), fibrinogen, interleukin-6 (IL-6), tumor necrosis factor-alpha (TNF-α), cortisol, total cholesterol, LDL-cholesterol, HDL-cholesterol, and triglycerides (Table 2).

### 2.5. Statistical Analysis

All the data from the study were analyzed using IBM SPSS Statistics 25 and illustrated using Microsoft Office Excel/Word 2024. Qualitative variables were written as counts or percentages and were tested between groups using Fisher’s exact test. Z-tests with Bonferroni correction were used to further detail the results obtained in the contingency tables.

Quantitative variables were written as means with standard deviations or medians with interquartile ranges. Normality of the quantitative variables was assessed using the Shapiro–Wilk test. Quantitative independent variables with non-parametric distribution were tested between groups using the Mann–Whitney U test. Quantitative related variables with non-parametric distribution were tested between measurements using the Friedman test (along with post hoc Dunn–Bonferroni tests). The threshold for the significance level for all tests was considered to be α = 0.05.

## 3. Results

### Data Analysis

Data from Table 3 shows the characteristics of the analyzed patients. The results show the following:

- Average age was 49.13 ± 7.1 years, with a median of 49 years (IQR = 45–54);
- A total of 56.5% of the patients were men, and most of them were living in an urban environment (63.8%);
- Average Hamilton score was 11.64 ± 9.38 points, median = 7 points (IQR = 4–19);
- A total of 49.3% of the patients had depression (54 patients were women (45%) while 82 patients were men (52.6%); with no significant differences according to gender—*p* = 0.226), and 15.9% of them had very severe depression;
- A total of 21% of the patients had acute coronary syndrome, most of them having N-STEMI (9.9%) or STEMI (11.2%);
- Average total cholesterol was 190.1 ± 44.08, median = 170 (IQR = 150–225);
- Average LDL-cholesterol was 122.81 ± 38.97, median = 109.5 (IQR = 92–153);
- Average HDL-cholesterol was 41.55 ± 2.72, median = 40 (IQR = 40–44);
- Average triglycerides level was 128.6 ± 39.72, median = 120 (IQR = 90–165).

**Table 3:** Characteristics of the analyzed patients.

Parameter	Value
Age (mean ± SD, median (IQR))	49.13 ± 7.1, 49 (45–54)
Gender (male) (*n*, %)	156 (56.5%)
Environment (urban) (*n*, %)	176 (63.8%)
Hamilton score (mean ± SD, median (IQR))	11.64 ± 9.38, 7 (4–19)
Depression (*n*, %)	136 (49.3%)
Depression grade (*n*, %)	
Absent	140 (50.7%)
Mild	32 (11.6%)
Moderate	32 (11.6%)
Severe	28 (10.1%)
Very severe	44 (15.9%)
Acute coronary syndrome (*n*, %)	58 (21%)
Acute coronary syndrome—type (*n*, %)	
Absent	198 (71.7%)
Unstable angina	20 (7.2%)
N-STEMI	27 (9.9%)
STEMI	31 (11.2%)
Total cholesterol (mean ± SD, median (IQR))	190.1 ± 44.08, 170 (150–225)
LDL-cholesterol (mean ± SD, median (IQR))	122.81 ± 38.97, 109.5 (92–153)
HDL-cholesterol (mean ± SD, median (IQR))	41.55 ± 2.72, 40 (40–44)
Triglycerides (mean ± SD, median (IQR))	128.6 ± 39.72, 120 (90–165)

**Figure 1:**
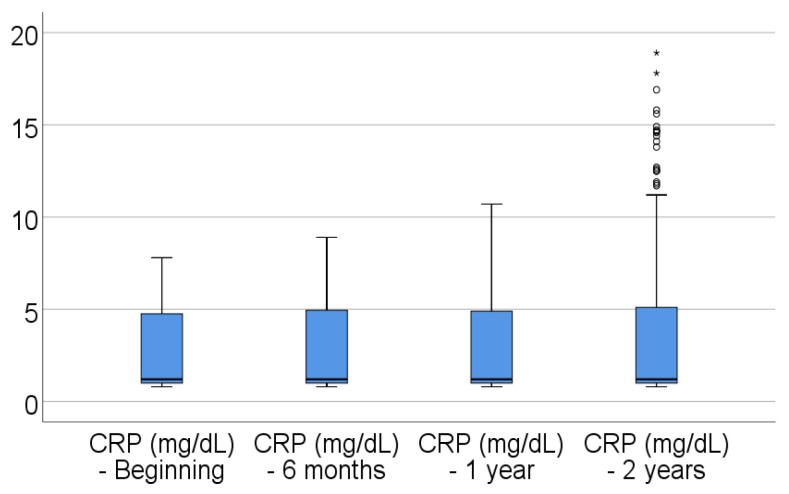
Evolution of CRP in the analyzed patients.

**Figure 2:**
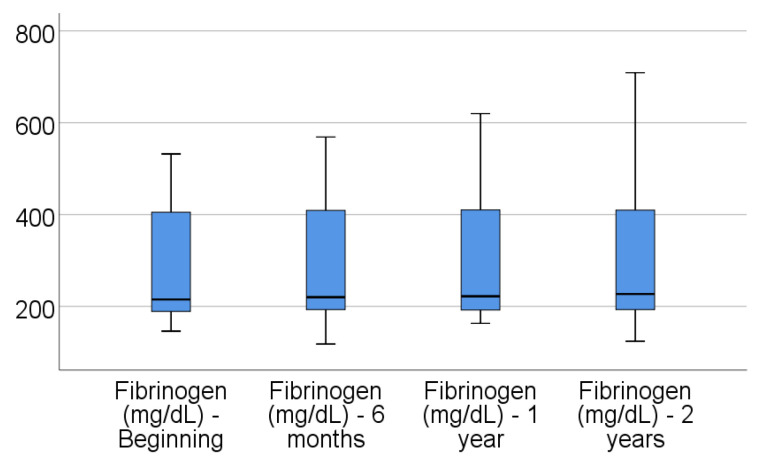
Evolution of fibrinogen in the analyzed patients.

**Figure 3:**
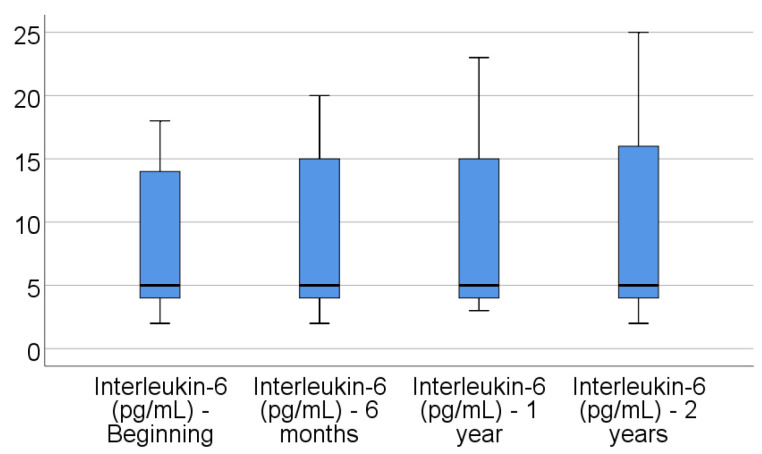
Evolution of interleukin-6 in the analyzed patients.

**Figure 4:**
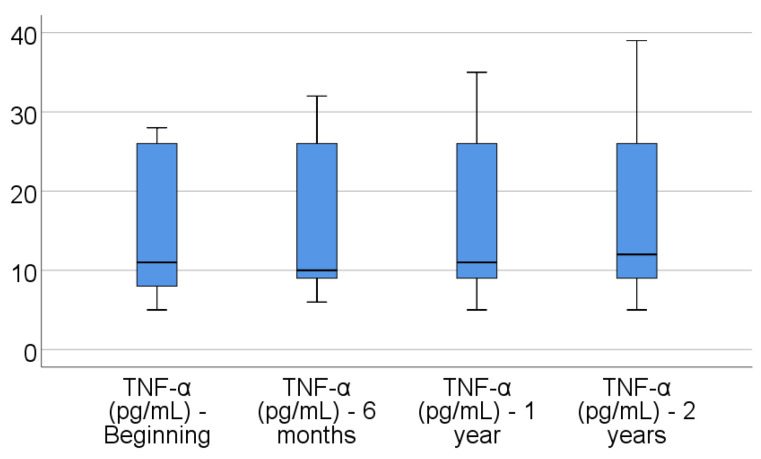
Evolution of TNF- α in the analyzed patients.

**Figure 5:**
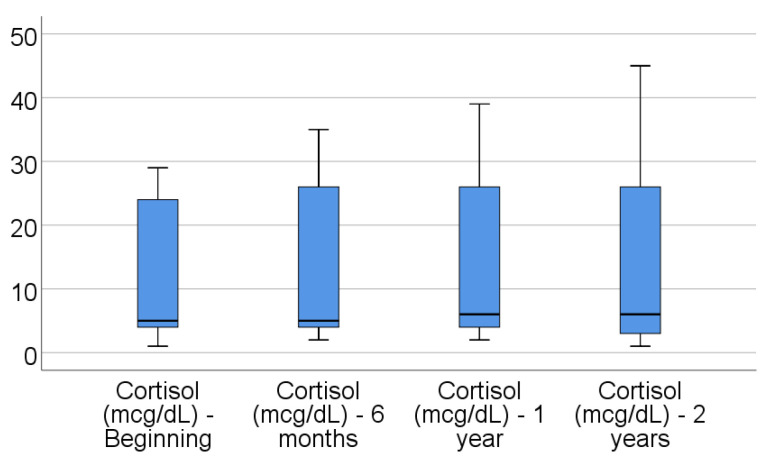
Evolution of cortisol in the analyzed patients.

Data from Table 4 and Figure 1, Figure 2, Figure 3, Figure 4 and Figure 5 show the evolution of inflammatory biomarkers in the analyzed patients. For all inflammatory biomarkers, there were observed significant differences between measurements (*p* < 0.001), showing, in general, increasing levels for all biomarkers after each visit; as such, the following significant differences were observed:

- CRP at the beginning was significantly lower compared to 6 months (*p* = 0.003), 1 year (*p* < 0.001), and 2 years (*p* < 0.001); while at 6 months, it was significantly lower compared to 1 year (*p* = 0.013) or 2 years (*p* < 0.001).
- Fibrinogen at the beginning was significantly lower compared to 6 months (*p* < 0.001), 1 year (*p* < 0.001), and 2 years (*p* < 0.001); while at 6 months, it was significantly lower compared to 2 years (*p* < 0.001).
- Interleukin-6 at the beginning was significantly lower compared to 6 months (*p* = 0.001), 1 year (*p* < 0.001), and 2 years (*p* < 0.001); while at 6 months, it was significantly lower compared to 2 years (*p* = 0.010).
- TNF-α at the beginning was significantly lower compared to 6 months (*p* < 0.001), 1 year (*p* < 0.001), and 2 years (*p* < 0.001); at 6 months, it was significantly lower compared to 1 year (*p* = 0.002) or 2 years (*p* < 0.001), and at 1 year, it was significantly lower than at 2 years (*p* = 0.012).
- Cortisol at the beginning was significantly lower compared to 6 months (*p* = 0.001), 1 year (*p* < 0.001), and 2 years (*p* < 0.001); while at 6 months, it was significantly lower compared to 2 years (*p* = 0.037).

Although the observed effect sizes are small (Kendall’s W = 0.126, 0.090, 0.070, 0.190, 0.096), the statistical power of the comparisons appears adequate, suggesting that the sample is representative of the target population.

**Table 4:** Evolution of inflammatory biomarkers in the analyzed patients.

**CRP**	**Mean ± SD**	**Median (IQR)**	**Mean Rank**	*p* *
Beginning	2.24 ± 1.96	1.2 (1–5)	1.99	<0.001 Kendall’s W = 0.126
6 months	2.36 ± 2.17	1.2 (1–5.1)	2.37	
1 year	2.6 ± 2.68	1.2 (1–5.1)	2.71	
2 years	3.07 ± 3.81	1.2 (1–5.1)	2.92	
**Post hoc Dunn–Bonferroni comparisons**				
CRP	Beginning	6 months	1 year	2 years
Beginning	-	0.003	<0.001	<0.001
6 months	0.003	-	0.013	<0.001
1 year	<0.001	0.013	-	0.332
2 years	<0.001	<0.001	0.332	-
**Fibrinogen**	**Mean ± SD**	**Median (IQR)**	**Mean Rank**	*p* *
Beginning	268.07 ± 106.6	215 (189–405)	2.00	<0.001 Kendall’s W = 0.090
6 months	278.93 ± 118.76	220 (193–409)	2.44	
1 year	288 ± 132.78	222 (192–410)	2.65	
2 years	299.35 ± 150.8	227 (193–409.75)	2.91	
**Post hoc Dunn–Bonferroni comparisons**				
Fibrinogen	Beginning	6 months	1 year	2 years
Beginning	-	<0.001	<0.001	<0.001
6 months	<0.001	-	0.323	<0.001
1 year	<0.001	0.323	-	0.092
2 years	<0.001	<0.001	0.092	-
**Interleukin-6**	**Mean ± SD**	**Median (IQR)**	**Mean Rank**	*p* *
Beginning	7.67 ± 5.41	5 (4–14)	2.07	<0.001 Kendall’s W = 0.070
6 months	8.05 ± 5.77	5 (4–15)	2.50	
1 year	8.47 ± 6.37	5 (4–15)	2.59	
2 years	9.17 ± 7.1	5 (4–16)	2.84	
**Post hoc Dunn–Bonferroni comparisons**				
Interleukin-6	Beginning	6 months	1 year	2 years
Beginning	-	0.001	<0.001	<0.001
6 months	0.001	-	1.000	0.010
1 year	<0.001	1.000	-	0.150
2 years	<0.001	0.010	0.150	-
**TNF-α**	**Mean ± SD**	**Median (IQR)**	**Mean Rank**	*p* *
Beginning	14.22 ± 7.93	11 (8–26)	1.82	<0.001 Kendall’s W = 0.190
6 months	14.97 ± 8.48	10 (9–26)	2.35	
1 year	15.8 ± 9.34	11 (9–26)	2.74	
2 years	16.53 ± 10.45	12 (9–26)	3.08	
**Post hoc Dunn–Bonferroni comparisons**				
TNF-α	Beginning	6 months	1 year	2 years
Beginning	-	<0.001	<0.001	<0.001
6 months	<0.001	-	0.002	<0.001
1 year	<0.001	0.002	-	0.012
2 years	<0.001	<0.001	0.012	-
**Cortisol**	**Mean ± SD**	**Median (IQR)**	**Mean Rank**	*p* *
Beginning	10.48 ± 9.84	5 (4–24)	1.98	<0.001 Kendall’s W = 0.096
6 months	11.78 ± 10.61	5 (4–26)	2.49	
1 year	12.96 ± 11.56	6 (4–26)	2.73	
2 years	13.2 ± 12.52	6 (3–26)	2.80	
**Post hoc Dunn–Bonferroni comparisons**				
Cortisol	Beginning	6 months	1 year	2 years
Beginning	-	<0.001	<0.001	<0.001
6 months	<0.001	-	0.185	0.037
1 year	<0.001	0.185	-	1.000
2 years	<0.001	0.037	1.000	-

* Related-samples Friedman’s two-way analysis of variance by ranks.

**Figure 6:**
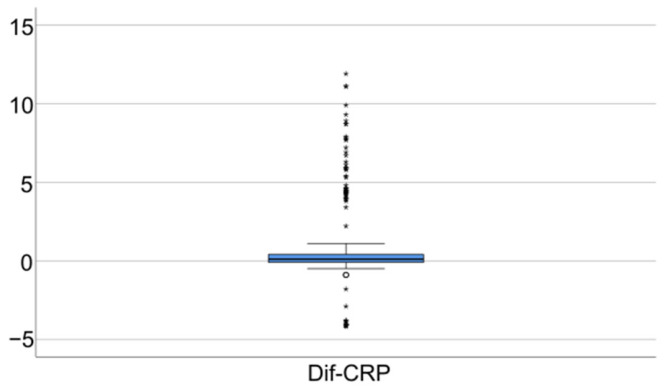
Difference of CRP from the beginning to 2 years of measurement.

**Figure 7:**
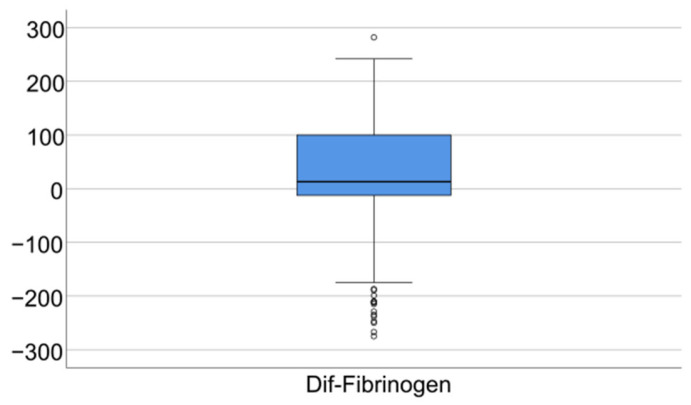
Difference of fibrinogen from the beginning to 2 years of measurement.

**Figure 8:**
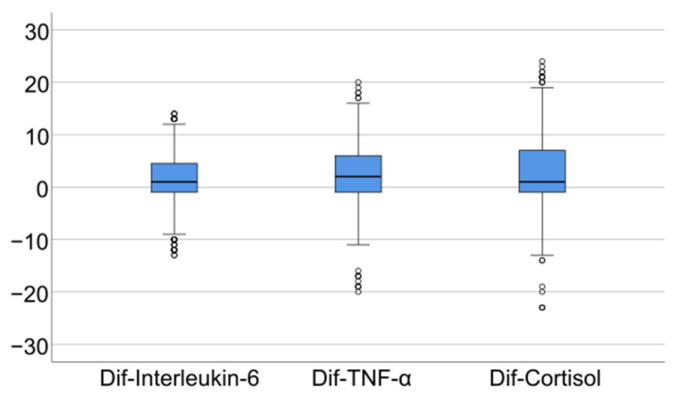
Difference of interleukin-6, TNF- α and cortisol from the beginning to 2 years of measurement.

Data from Table 5 and Figure 6, Figure 7 and Figure 8 show the differences in the evolution of inflammatory parameters between the baseline value and 2-year measurements. According to the data presented above, all the parameters had their highest value at the second-year measurement as the largest difference in the evolution would be measured between the baseline value and the second-year measurement. The results show the following increases observed at 2 years for each of the analyzed parameters:

- CRP: mean = 0.83 ± 2.62 (median = 0.1, IQR = −0.1–0.4);
- Fibrinogen: mean = 31.41 ± 103.48 (median = 13, IQR = −13–100);
- Interleukin-6: mean = 1.5 ± 5.48 (median = 1, IQR = −1–5);
- TNF-α: mean = 2.32 ± 6.06 (median = 2, IQR = −1–6);
- Cortisol: mean = 2.73 ± 7.19 (median = 1, IQR = −1–7).

**Table 5:** Differences in evolution of inflammatory parameters between the baseline value and 2-year measurements.

Dif. Beginning/2 Year	Mean ± SD	Median (IQR)	Min	Max
CRP	0.83 ± 2.62	0.1 (−0.1–0.4)	−4	12
Fibrinogen	31.41 ± 103.48	13 (−13–100)	−275	282
Interleukin-6	1.5 ± 5.48	1 (−1–5)	−13	14
TNF-α	2.32 ± 6.06	2 (−1–6)	−20	20
Cortisol	2.73 ± 7.19	1 (−1–7)	−23	24

**Figure 9:**
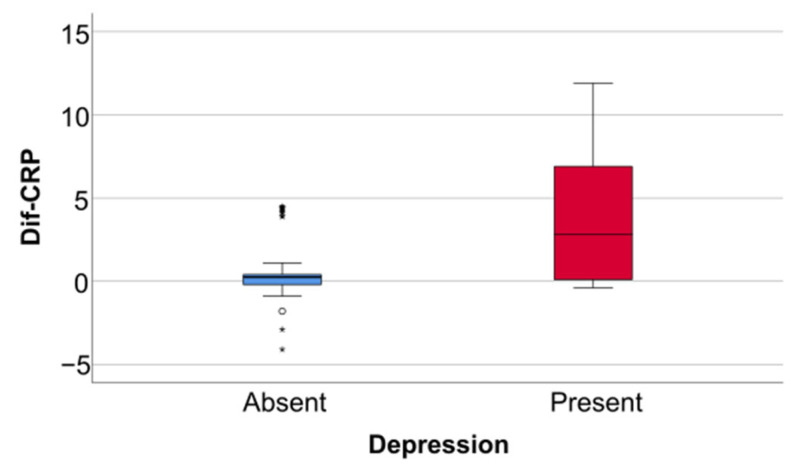
A comparison of the CRP difference from the beginning to 2 years in women according to the existence of depression.

**Figure 10:**
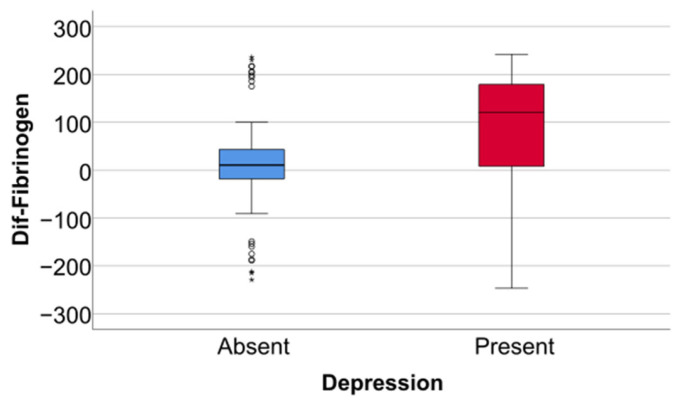
A comparison of fibrinogen differences from the beginning to 2 years in women according to the existence of depression.

**Figure 11:**
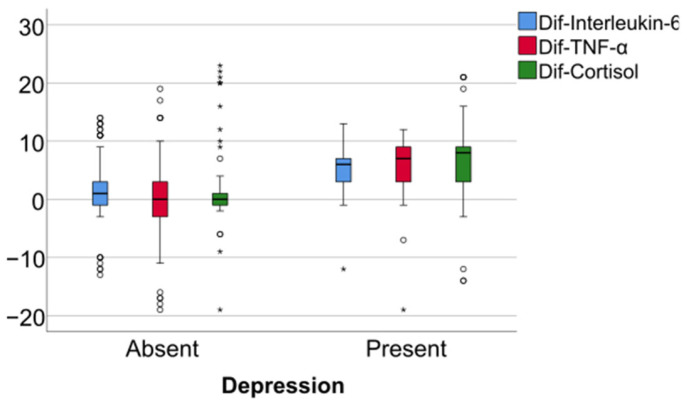
A comparison of interleukin-6, TNF-alpha, and cortisol differences from beginning to 2 years in women according to the existence of depression.

Data from Table 6 and Figure 9, Figure 10 and Figure 11 show the comparison of inflammatory differences from baseline to 2 years in women according to the existence of depression. Based on the Z scores obtained in each Mann–Whitney U test, a rank-biserial correlation (r_rb_) was calculated to estimate the effect size in each comparison. Also, a value for Cohen’s d was calculated based on r_rb_ to calculate the post hoc power of each comparison. As seen in both r_rb_ values and *d* values, the effect size in all comparisons was large.

**Figure 12:**
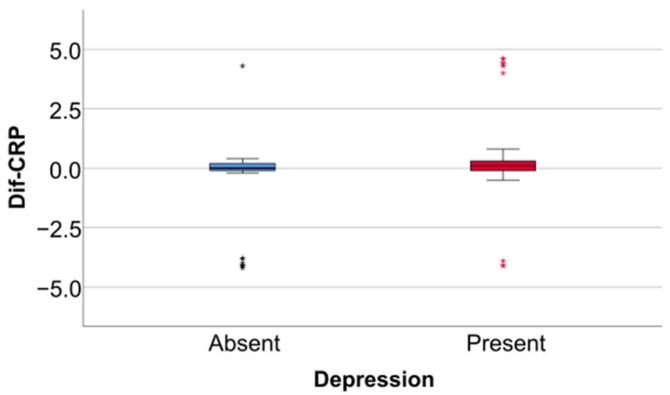
A comparison of the CRP difference from the beginning to 2 years in men according to the existence of depression.

**Figure 13:**
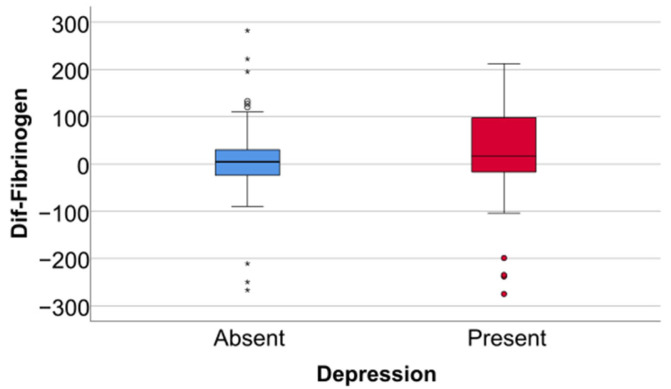
A comparison of fibrinogen differences from the beginning to 2 years in men according to the existence of depression.

**Figure 14:**
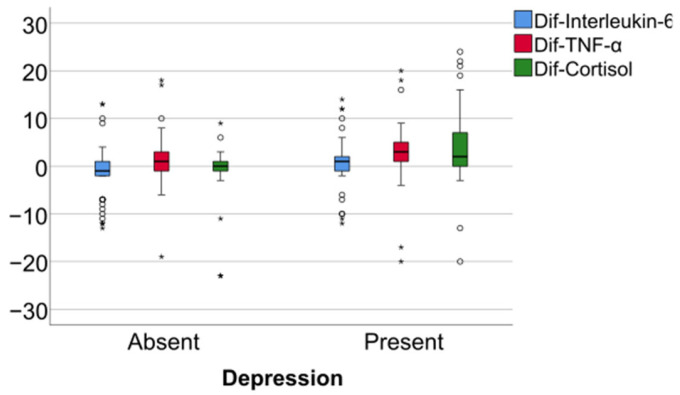
A comparison of interleukin-6, TNF-alpha, and cortisol differences from beginning to 2 years in men according to the existence of depression.

Data from Table 7 and Figure 12, Figure 13 and Figure 14 show the comparison of inflammatory differences from baseline to 2 years in men according to the existence of depression. Results show that patients with depression had significantly higher increases at 2 years compared to baseline for all investigated parameters (CRP—*p* = 0.032/Fibrinogen—*p* = 0.030/Interleukin-6—*p* < 0.001/TNF-α -*p* = 0.007/Cortisol—*p* < 0.001).

**Figure 15:**
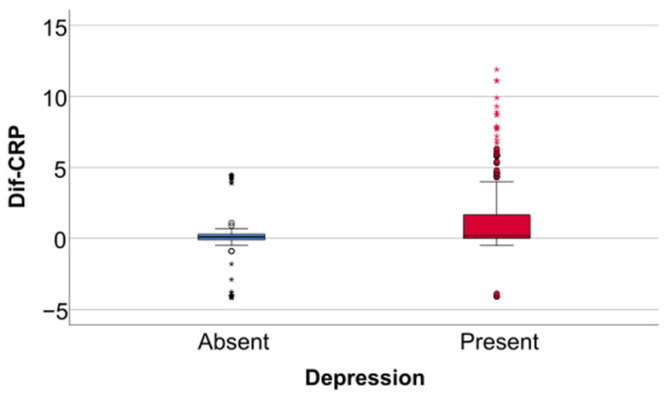
A comparison of the CRP difference from the beginning to 2 years according to the existence of depression.

**Figure 16:**
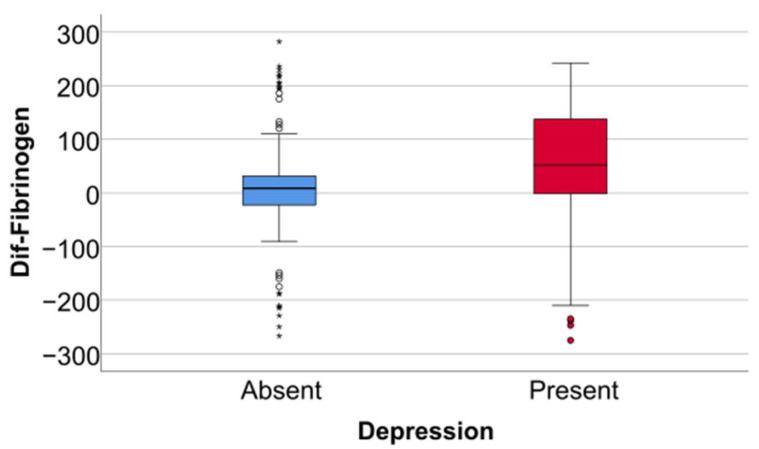
A comparison of fibrinogen differences from the beginning to 2 years according to the existence of depression.

**Figure 17:**
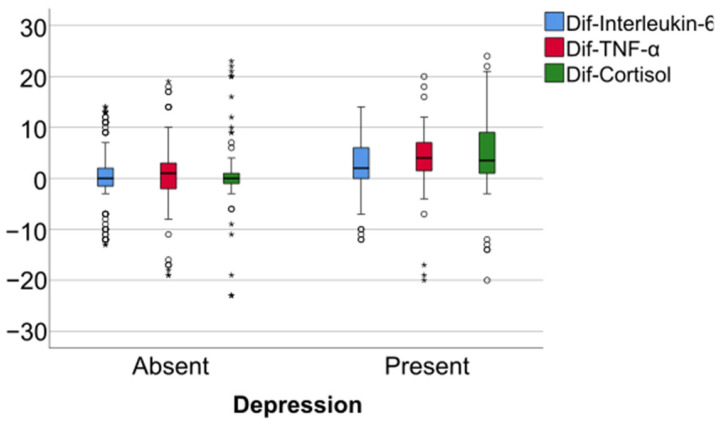
A comparison of interleukin-6, TNF-alpha, and cortisol differences from the beginning to 2 years according to the existence of depression.

Data from Table 8 and Figure 15, Figure 16 and Figure 17 show the comparison of inflammatory differences from baseline to 2 years according to the existence of depression. Results show that patients with depression had significantly higher increases at 2 years compared to baseline for all investigated parameters (CRP—*p* = 0.001/Fibrinogen—*p* < 0.001/Interleukin-6—*p* < 0.001/TNF-α -*p* < 0.001/Cortisol—*p* < 0.001).

**Figure 18:**
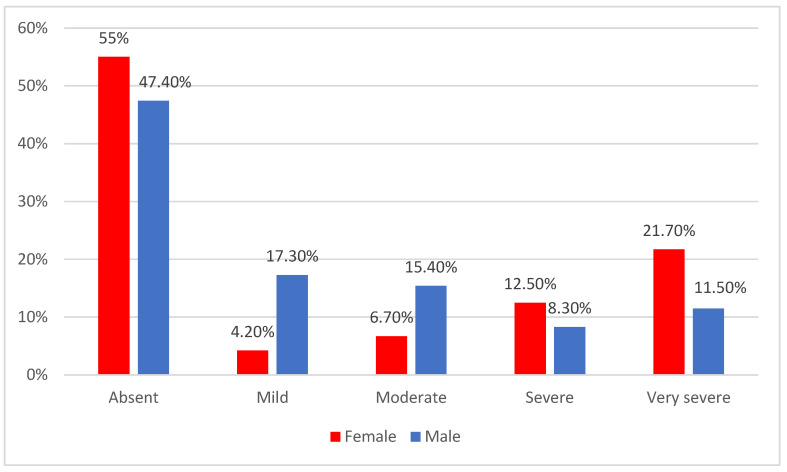
The distribution of the patients according to gender and depression grade.

Data from Table 9 and Figure 18 show the distribution of the patients according to gender and depression grade. Differences were significant according to Fisher’s exact test (*p* < 0.001), and Z-tests with Bonferroni correction showed that among patients with mild depression (17.3% vs. 4.2%) or moderate depression (15.4% vs. 6.7%), there were significantly more men than women; while among patients with very severe depression, there were significantly more women than men (21.7% vs. 11.5%).

**Figure 19:**
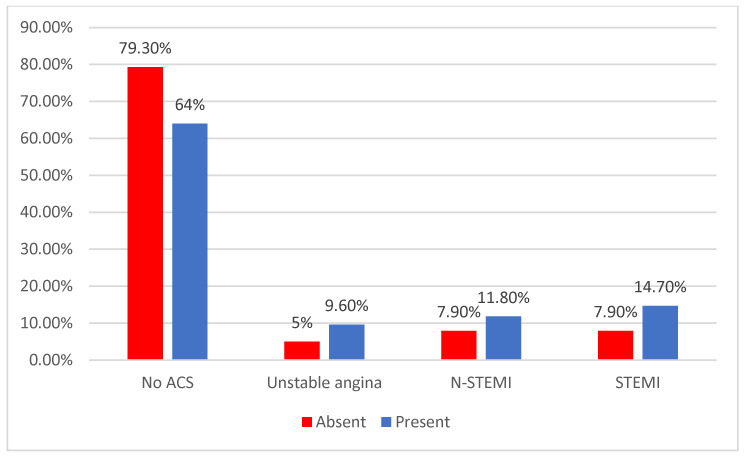
The distribution of the patients according to the existence of depression and the acute coronary syndrome type.

Data from Table 10 and Figure 19 show the distribution of the patients according to the existence of depression and the type and presence of the acute coronary syndrome. Differences were significant according to Fisher’s exact tests (*p* = 0.038/*p* = 0.043), and Z-tests with Bonferroni correction showed only that patients with no acute coronary syndrome were significantly less associated with depression (79.3% vs. 64%, *p* = 0.043), while in the other association, it has been shown that patients with any type of acute coronary syndrome were significantly more associated with depression (26.5% vs. 15.7%, *p* = 0.038).

**Figure 20:**
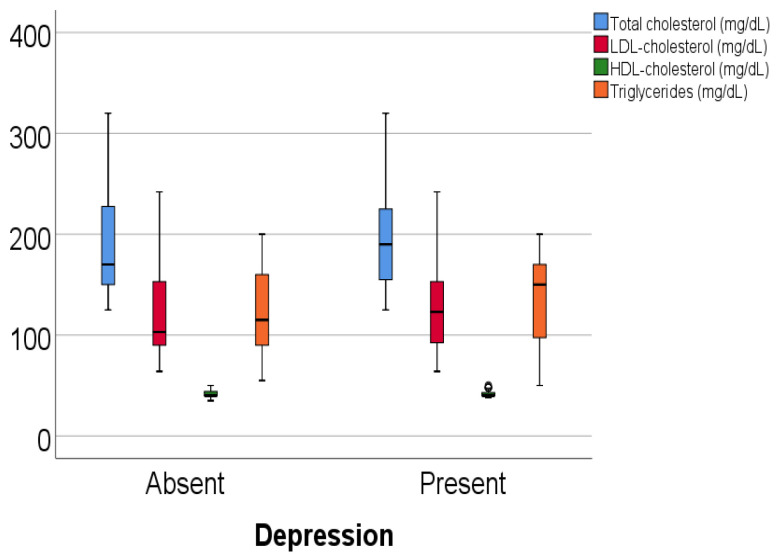
A comparison of lipid panel parameters according to the existence of depression.

Data from Table 11 and Figure 20 show the comparison of lipid panel parameters according to the existence of depression. Results show none of the lipid panel parameters were significantly different between patients with or without depression (total cholesterol—*p* = 0.363, LDL-cholesterol—*p* = 0.392, HDL-cholesterol—*p* = 0.131, triglycerides—*p* = 0.141).

## 4. Discussion

This research consistently shows that individuals diagnosed with depression are at a substantially increased risk of developing an acute coronary syndrome (ACS) compared to those without depression. To reduce the likelihood of misclassifying somatic symptoms commonly seen in cardiovascular disease—such as fatigue or disturbed sleep—as depressive symptoms, our study utilized a two-pronged diagnostic approach. We combined the Structured Clinical Interview for DSM Disorders (SCID) with the Hamilton Depression Rating Scale (HDRS) [8,10,11]. Employing both clinician-administered interviews and symptom rating tools enhanced the internal validity and homogeneity of our depression cohort, thereby strengthening the reliability of associations found between depressive symptoms and inflammatory biomarkers. Depression is a well-recognized independent risk factor for cardiovascular disease (CVD), including acute myocardial infarction (AMI) [12,13]. While inflammation is a necessary part of the healing process following cardiac events, an exaggerated or prolonged inflammatory response can contribute to adverse outcomes such as heart failure and ventricular remodeling [14,15,16].

Our findings indicate that depressed women exhibited significantly elevated levels of inflammatory markers over a two-year period. This included increases in C-reactive protein (CRP), fibrinogen, interleukin-6 (IL-6), tumor necrosis factor-alpha (TNF-α), and cortisol (all *p* < 0.001). Gender differences in depression severity were also noted: men were more commonly represented in the mild to moderate depression categories, while women predominated in the severe depression group. This finding aligns with existing epidemiological data showing higher lifetime rates of depression in women [17]. Several factors may contribute to this difference, including the anti-inflammatory role of estrogen [18], heightened immune sensitivity in females [19], and sociocultural stressors such as caregiving roles or unequal access to mental health services [20]. An extensive study involving more than 4 million participants reported that depression elevates the risk of cardiovascular disease in both men and women; however, the effect appears to be more significant in women. The hazard ratio for CVD was 1.64 among women with depression, compared to 1.39 in men, indicating a potentially greater cardiovascular burden associated with depression in females [21]. Evidence suggests that the intensity and clinical presentation of depression can affect cardiovascular outcomes in sex-specific ways. According to a meta-analysis, women with coronary artery disease are nearly twice as likely as men to experience major depression, implying that more severe depressive profiles may be particularly prevalent among females with coronary artery disease [22].

Our study found that participants with severe depressive symptoms had significantly higher concentrations of CRP, IL-6, and TNF-α, reaffirming a robust link between inflammation and depression. A thorough meta-analysis established a clear link between increased concentrations of IL-6 and CRP and the presence of major depressive disorder. The authors highlighted the importance of additional research to better understand the underlying mechanisms and potential treatment strategies related to this association [23]. Another meta-analysis reported that individuals with depression exhibited notably elevated blood levels of CRP, IL-6, and TNF-α relative to healthy controls, with effect sizes ranging from moderate to large (0.54 to 1.97). These results remained consistent across multiple sensitivity analyses, underscoring the strength of the association [24].

The reciprocal relationship between depression and inflammation carries important consequences for both preventive strategies and therapeutic approaches. Inflammatory cytokines can alter monoaminergic transmission and reduce brain-derived neurotrophic factor (BDNF) levels, further impairing emotional and cognitive functioning [25,26,27,28]. Depression may exacerbate inflammation via stress-related hypothalamic–pituitary–adrenal (HPA) axis activation, autonomic imbalance, and unhealthy lifestyle habits such as poor diet, sedentary behavior, and substance use [29,30,31,32]. This feedback loop not only sustains the depressive state but also accelerates cardiovascular decline. Given this two-way relationship, anti-inflammatory interventions—including NSAIDs, cytokine inhibitors, and omega-3 fatty acids—have shown promise in mitigating depressive symptoms, especially in patients with elevated baseline inflammation [33,34]. Additionally, treating depression with pharmacologic or psychological therapies has been associated with reduced systemic inflammation, supporting the notion that mood and immune regulation are interlinked [35].

In parallel, depressed men in our study also showed significant increases in the same inflammatory biomarkers over two years (e.g., CRP *p* = 0.001; IL-6, TNF-α, fibrinogen, and cortisol all *p* < 0.001). These findings resonate with the Prospective Epidemiological Study of Myocardial Infarction, where Empana et al. (2005) found that CRP and IL-6 levels were independently associated with cardiovascular events in depressed men, even after adjusting for traditional coronary risk factors [36]. It is known that AMI triggers a sustained rise in inflammatory markers, including cytokines and acute-phase proteins, which can persist for days or weeks post-event [29,37,38,39]. However, causality remains uncertain. Depression might precede inflammatory activation, or conversely, early vascular changes may elicit depressive symptoms through cytokine-mediated pathways. Longitudinal mediation analyses and animal models are needed to resolve these temporal ambiguities. In our study, men were over-represented in cases of mild and moderate depression, whereas women more frequently fell into the severe category (e.g., 21.7% of women had very severe depression vs. 11.5% of men). This pattern aligns with previous studies such as CARDIO2000, which found higher depressive symptom prevalence in women, although men tend to experience AMI earlier and more frequently [40,41,42]. Findings regarding gender-specific CVD risk are mixed: some studies report higher ACS risk in depressed women [43,44], others show equal CHD risk across genders [45,46,47,48], and yet, others suggest greater ischemic heart disease mortality in depressed men [49].

Emerging evidence also indicates that racial differences may influence the relationship between depression and CVD. For instance, depressive symptoms may pose a higher cardiovascular risk in African American populations compared to white individuals [50,51]. Our study, limited to Romanian participants, may not fully capture these complex sociodemographic dynamics. The ethnic, cultural, and healthcare-related factors unique to different global populations—such as those in South Asia or sub-Saharan Africa—may shape how depression and cardiovascular conditions interact [52,53,54,55,56]. Estimates suggest that the lifetime prevalence of major depression ranges from 8% to 12%, with women affected more frequently than men [57,58]. Many cases remain undiagnosed or untreated, contributing to chronic illness and recurrent episodes [57,59]. Among individuals with existing CVD, approximately 15–22% also experience depressive disorders [43]. Depression has been linked to a 2–4-fold increase in mortality following a myocardial infarction [46], particularly when symptoms are persistent or severe [44,46]. While past studies have associated depression with lipid abnormalities, these relationships may have been obscured in our study by uncontrolled confounders such as statin use, diet, and exercise, which were not consistently recorded. Future research should account for these factors when exploring the depression–lipid–CVD axis.

Our study is strengthened by its inclusion of both male and female participants and its longitudinal tracking of multiple inflammatory markers. This design allowed for a nuanced understanding of sex-based differences and dynamic changes in biomarker levels. Despite the valuable insights gained, several limitations should be acknowledged. First, the retrospective observational design inherently carries a risk of selection bias as participants were not randomly assigned and data were collected from the hospital database rather than through prospective tracking. This limits our ability to establish causality and increases the possibility of unmeasured confounding factors. Second, although we made efforts to include a demographically representative sample, the absence of randomization and potential confounders—such as variations in socioeconomic status and the presence of comorbidities—may affect the external validity of our findings. These factors could influence both depression severity and cardiovascular outcomes, thereby limiting the generalizability of our results to broader populations. Lastly, the sample size, while sufficient to detect meaningful trends in our primary outcomes, remains relatively small compared to larger population-based cohort studies.

## 5. Conclusions

This study highlights a strong association between depressive symptoms and elevated inflammatory markers, pointing to a potential biological link between mental health and increased cardiovascular risk. While the retrospective nature of the study limits conclusions about causality, the findings suggest that monitoring inflammation in individuals with depression may improve cardiovascular risk assessment. Clinically, these results support integrating mental health screening into cardiovascular care. Routine depression assessments, alongside evaluation of inflammatory markers, may help identify patients at higher risk for cardiovascular events earlier. Treatment approaches could also benefit from considering the anti-inflammatory properties of some antidepressants, such as selective serotonin reuptake inhibitors (SSRIs), as well as lifestyle modifications that reduce inflammation—like regular exercise, healthier diets, and stress management. Future research should prioritize longitudinal studies to better understand the direction and nature of the relationship between depression, inflammation, and cardiovascular outcomes. Exploring integrated treatment strategies that address both mental and physical health may offer more effective care for patients with comorbid conditions. Additionally, CRP and IL-6 show potential as practical biomarkers for cardiovascular risk stratification in depressed individuals, though further research is needed to confirm their utility and determine how best to apply them in clinical settings. Studies examining specific pathways, such as IL-6’s role in endothelial dysfunction, could also uncover new therapeutic targets. Overall, these findings support a multidisciplinary approach that bridges mental and physical health to improve outcomes in patients with both depression and cardiovascular disease.

## Figures and Tables

**Table 1:** Baseline characteristics of the participants included in the study.

Divided by:	The Depression Group—136 Patients		The Control Group—140 Patients	
Gender	Men—82 (60.30%)	Women—54 (39.70%)	Men—74 (52.85%)	Women—66 (47.15%)
Provenience	Urban—92 (59%)	Rural—36 (30%)	Rural—64 (41%)	Urban—84 (70%)
Age	between 33 and 67 years		between 34 and 68 years	

**Table 2:** Normal laboratory reference ranges for men and women [9].

Laboratory Analyses	Normal Values—Men	Normal Values—Women
CRP	0.8–1 mg/dL	1–1.3 mg/dL
Fibrinogen	200–400 mg/dL	200–400 mg/dL
IL-6	5–15 pg/mL	5–15 pg/mL
TNF-α	0–24.47 pg/mL	0–24.47 pg/mL
Cortisol	5–25 mcg/dL	5–25 mcg/dL
Total Cholesterol	<200 mg/dL	<200 mg/dL
Ldl-Cholesterol	<100 mg/dL	<100 mg/dL
Hdl-Cholesterol	<40 mg/dL	<50 mg/dL
Triglycerides	<150 mg/dL	<150 mg/dL

**Table 6:** Comparisons of inflammatory differences from baseline to 2 years in women according to the existence of depression.

**Depression/CRP**	**Mean ± SD**	**Median (IQR)**	**Mean Rank**	*p* *
Absent (*n* = 66)	0.53 ± 1.66	0.25 (−0.2–0.4)	49.13	<0.001 Z = −3.97 r_rb_ = −0.724 d = −2.104
Present (*n* = 54)	3.7 ± 3.88	2.8 (0.1–6.98)	74.4	
**Depression/Fibrinogen**	**Mean ± SD**	**Median (IQR)**	**Mean Rank**	*p* *
Absent (*n* = 66)	13.48 ± 110.5	10.5 (−18–43.5)	49.61	<0.001 Z = −3.793 r_rb_ = −0.692 d = −1.919
Present (*n* = 54)	98.96 ± 104.76	121 (7.25–180)	73.81	
**Depression/IL-6**	**Mean** ± **SD**	**Median (IQR)**	**Mean Rank**	*p* *
Absent (*n* = 66)	1.76 ± 6.51	1 (−1–3.25)	49.13	<0.001 Z = −3.972 r_rb_ = −0.725 d = −2.106
Present (*n* = 54)	5.39 ± 4.25	6 (3–7.25)	74.40	
**Depression/TNF-α**	**Mean** ± **SD**	**Median (IQR)**	**Mean Rank**	*p* *
Absent (*n* = 66)	0.17 ± 7.52	0 (−3–3)	45.72	<0.001 Z = −5.158 r_rb_ = −0.941 d = −5.598
Present (*n* = 54)	5.59 ± 5.09	7 (2.75–9)	78.56	
**Depression/Cortisol**	**Mean** ± **SD**	**Median (IQR)**	**Mean Rank**	*p* *
Absent (*n* = 66)	1.98 ± 7.46	0 (−1–1.25)	47.55	<0.001 Z = −4.535 r_rb_ = −0.827 d = −2.953
Present (*n* = 54)	6.87 ± 7.38	8 (3–9.25)	76.33	

* Mann–Whitney U test.

**Table 7:** A comparison of inflammatory differences from baseline to 2 years in men according to the existence of depression.

**Depression/CRP**	**Mean ± SD**	**Median (IQR)**	**Mean Rank**	*p* *
Absent	−0.38 ± 1.47	0 (−0.1–0.2)	69.91	0.032
Present	0.26 ± 1.42	0.1 (−0.1–0.3)	85.20	
**Depression/Fibrinogen**	**Mean** ± **SD**	**Median (IQR)**	**Mean Rank**	*p* *
Absent	3.43 ± 83.34	4.5 (−24.75–30)	70.24	0.030
Present	26.16 ± 94.63	17 (−18–98.5)	85.95	
**Depression/IL-6**	**Mean** ± **SD**	**Median (IQR)**	**Mean Rank**	*p* *
Absent	−1 ± 4.87	−1 (−2–1)	64.61	<0.001
Present	0.98 ± 4.27	1 (−1–2.25)	91.03	
**Depression/TNF-α**	**Mean** ± **SD**	**Median (IQR)**	**Mean Rank**	*p* *
Absent	1.39 ± 4.77	1 (−1.25–3)	68.30	0.007
Present	2.72 ± 5.42	3 (1–5)	87.70	
**Depression/Cortisol**	**Mean** ± **SD**	**Median (IQR)**	**Mean Rank**	*p* *
Absent	−0.91 ± 5.19	0 (−1–1.25)	59.31	<0.001
Present	3.77 ± 6.8	2 (0–7)	95.82	

* Mann–Whitney U test.

**Table 8:** A comparison of inflammatory differences from baseline to 2 years according to the existence of depression.

**Depression/CRP**	**Mean ± SD**	**Median (IQR)**	**Mean Rank**	*p* *
Absent	0.05 ± 1.62	0.1 (−0.1–0.3)	122.18	0.001
Present	1.63 ± 3.16	0.2 (0–1.93)	154.17	
**Depression/Fibrinogen**	**Mean** ± **SD**	**Median (IQR)**	**Mean Rank**	*p* *
Absent	8.17 ± 96.86	8.5 (−22.7–32.2)	119.39	<0.001
Present	55.07 ± 104.7	52 (−1.75– 141.2)	158.17	
**Depression/IL-6**	**Mean** ± **SD**	**Median (IQR)**	**Mean Rank**	*p* *
Absent	0.3 ± 5.85	0 (−1.75–2)	116.53	<0.001
Present	2.73 ± 4.77	2 (0–6)	161.11	
**Depression/TNF-α**	**Mean** ± **SD**	**Median (IQR)**	**Mean Rank**	*p* *
Absent	0.81 ± 6.23	1 (−2–3)	111.45	<0.001
Present	3.86 ± 5.46	4 (1.25–7)	166.35	
**Depression/Cortisol**	**Mean** ± **SD**	**Median (IQR)**	**Mean Rank**	*p* *
Absent	0.51 ± 6.5	0 (−1–1)	105.82	<0.001
Present	5 ± 7.17	3.5 (1–9)	172.14	

* Mann–Whitney U test.

**Table 9:** The distribution of the patients according to gender and depression grade.

Gender/ Depression	Female		Male		*p* *
	*n*	%	*n*	%	
Absent	66	55%	74	47.4%	<0.001
Mild	5	4.2%	27	17.3%	
Moderate	8	6.7%	24	15.4%	
Severe	15	12.5%	13	8.3%	
Very severe	26	21.7%	18	11.5%	

* Fisher’s exact test.

**Table 10:** The distribution of the patients according to the existence of depression and the type and presence of the acute coronary syndrome.

**ACS/Depression**	**Absent**		**Present**		*p* *
	***n***	**%**	***n***	**%**	
No ACS	118	84.3%	100	73.5%	0.038
With ACS	22	15.7%	36	26.5%	
**ACS Type/Depression**	**Absent**		**Present**		*p* *
	***n***	**%**	***n***	**%**	
No ACS	111	79.3%	87	64%	0.043
Unstable angina	7	5%	13	9.6%	
N-STEMI	11	7.9%	16	11.8%	
STEMI	11	7.9%	20	14.7%	

* Fisher’s exact test.

**Table 11:** A comparison of lipid panel parameters according to the existence of depression.

**Depression/Total Cholesterol**	**Mean ± SD**	**Median (IQR)**	**Mean Rank**	*p* *
Absent	187.5 ± 44.6	170 (150–228.75)	134.20	0.363
Present	192.7 ± 43.5	190 (155–225)	142.93	
**Depression/LDL-C**	**Mean** ± **SD**	**Median (IQR)**	**Mean Rank**	*p* *
Absent	120.7 ± 39.5	103 (90–153)	134.45	0.392
Present	124.9 ± 38.4	123 (92.2–153)	142.67	
**Depression/HDL-C**	**Mean** ± **SD**	**Median (IQR)**	**Mean Rank**	*p* *
Absent	41.76 ± 2.83	40 (40–44)	145.45	0.131
Present	41.34 ± 2.6	40 (40–43)	131.34	
**Depression/Triglycerides**	**Mean** ± **SD**	**Median (IQR)**	**Mean Rank**	*p* *
Absent	125.07 ± 39.5	115 (90–160)	131.55	0.141
Present	132.2 ± 39.7	150 (96.25–170)	145.65	

* Mann–Whitney U test.

## Data Availability

All data used in this study are stored in the database of the Clinical County Emergency Hospital of Oradea, located in Bihor County, Romania.
